# Exosomes: Tiny Clues for Mast Cell Communication

**DOI:** 10.3389/fimmu.2015.00073

**Published:** 2015-02-18

**Authors:** Federica D’Incà, Carlo E. Pucillo

**Affiliations:** ^1^Department of Medical and Biological Sciences, University of Udine, Udine, Italy

**Keywords:** mast cell, exosomes, cell–cell communication

Intercellular communication is an essential bedrock in the maintenance and development of multicellular organisms.

Conventional models of cellular exchange include transfer of secreted molecules and direct contact; only recently, exosomes have emerged as a new avenue for cell-to-cell communication.

Discovered nearly 30 years ago, exosomes were initially considered little cellular garbage disposals acting to discard unwanted proteins and molecules, and research on exosome biology developed at an extremely slow pace; it is only in the past two decades or so that we have been able to unscramble some of the various biological roles of these nanovesicles.

The foundation for the hypothesis that exosomes could play a large and active role in intercellular communication came in 1996, when Graca Raposo published her discovery of exosomes, secreted by Epstein-Barr virus transformed B lymphocytes, able to induce antigen-specific MHC class II-restricted T cell responses (1). Interest in exosomes was boosted further by a publication in 1998 by Zitvogel’s team reporting that dendritic cell-derived exosomes could promote the induction of antitumor response in mice, prompting the first attempt for their clinical application (2). Over the past few years, however, considerable research really brings us closer to harnessing the potential of these tiny vesicles as both a diagnostic and therapeutic tool.

Secreted by multiple cell types and virtually found in all body fluids, exosomes are nano-sized, cell membrane surrounded structures harboring a broad range of biomolecules, including mRNAs, miRNAs, and proteins linked to cell type-associated functions. To understand the biological function of exosomes, a number of proteomic and transcriptomic profile studies has been performed. The huge amount of data obtained have allowed researchers to develop Exocarta.com, a free web-based and centralized repository of molecules that have been documented in exosomes, in order to facilitate novel biological insight. Exosomes were subsequently found to play important role in influencing physiological pathways in the recipient cells (3).

In this scenario, studies on MC-derived exosomes represented a fundamental starting point for the definition of their biological functions.

MCs have been slowly but definitely shed out their image of allergy-causing troublemakers and have emerged as versatile cells with the ability to orchestrate several biological processes. Even though MCs are traditionally considered a secretory cell type, it is possible to ascribe their phenotypic and functional plasticity not only to their capacity to respond to a wide range of signals with the production of distinct patterns of mediators, but also to their ability in establishing direct intercellular interactions (4). Nevertheless, in addition to these traditional mechanisms for cell-to-cell communication, exosomes may also empower MCs to interact with different targets.

In 2001, Skokos described for the first time, the ability of bone marrow MCs, peritoneal MCs, and MC lines P815 and MC/9 to constitutively secrete exosomes. More importantly, he provided that MCs use exosomes as sophisticated messengers showing immunoregulatory activity on B and T lymphocytes (5).

Six years later, the remarkable discovery by the group of Lötvall has provided a major breakthrough in exosome research opening up new avenues regarding diagnosis and therapeutics. They demonstrated that exosomes from human MCs carry a distinct repertoire of both mRNA and microRNA, also documenting the ability of MCs to shuttle RNA between each other via exosomes (6).

Since then, few evidence indicates that MC-derived exosomes play a pivotal role in cell-to-cell communication. The functional consequences of such vesicle transfers include the induction, amplification, and/or modulation of immune responses, epigenetic reprograming as well as the acquisition of new functional properties by recipient cells (tumor cells, muscle cells, and endothelial cells). Owing to the limited progress in the field, the pathophysiological relevance of exosomes released by MCs have been unaddressed. However, the strategic and ubiquitous tissue distribution of these cells and their ability to release different types of nanovesicles may provide them with an unique opportunity for harnessing and improving exosomes’ therapeutic and diagnostic capabilities.

With Lötvall’s discovery and the concomitant explosion of studies on microRNAs, the importance of exosomes has extended beyond their natural function and attempts to find practical application for exosomes are currently expanding. Therapeutically, exosomes may represent an efficient vehicle for drug or gene delivery with fewer potential hazards from a safety standpoint, because of their excellent host biodistribution and biocompatibility as well as their natural targeting properties.

Moreover, exosomes are garnering attention in diagnostics as cost-effective and efficient biomarkers. Since they can be found in most body fluids, exosomes can be collected in an easily and non-invasive way, making possible the real-time tracking of a patient’s disease progression (7).

As documented in clinicaltrial.gov, exosomes have already been approved for their use in clinical trials for different target patients. However, clinical translation is limited by the lack of scalable and economically viable methods for generation and loading of exosomes.

Undoubtedly, exosome-based therapy and diagnostics offer an exceptional alternative for diseases treatment in the future. Technological improvement of standardized purification method, a systematic characterization, an overall engineerization approach will help us to improve their utilization as diagnostic markers and further develop exosome-based translational nanomedicine.

The field of exosomics is as such still in its infancy, and consequently much basic research and integrative studies remain to be done. Certainly, a significant amount of work still lies ahead before the composition, function, and potential of these intriguing vesicles have been unrevealed.

## Conflict of Interest Statement

The authors declare that the research was conducted in the absence of any commercial or financial relationships that could be construed as a potential conflict of interest.
